# Effects of electrolyte supplementation on performance and physiological responses of preconditioning beef calves

**DOI:** 10.1093/tas/txae016

**Published:** 2024-02-09

**Authors:** Matheus F L Ferreira, Gracia P Hernandez, Aline C R Santos, David Bohnert, Nathan Upah, Juliana Ranches

**Affiliations:** Eastern Oregon Agricultural Research Center, Oregon State University, Burns, OR 97720, USA; Eastern Oregon Agricultural Research Center, Oregon State University, Burns, OR 97720, USA; Eastern Oregon Agricultural Research Center, Oregon State University, Burns, OR 97720, USA; Eastern Oregon Agricultural Research Center, Oregon State University, Burns, OR 97720, USA; TechMix LLC, Stewart, MN 55385, USA; Eastern Oregon Agricultural Research Center, Oregon State University, Burns, OR 97720, USA

**Keywords:** drinking, hydration, stress, water, weaning

## Abstract

The objective of this study was to evaluate the effects of electrolyte solution supplementation on the performance and physiological responses of beef calves during a 45-d preconditioning phase. Forty Angus × Hereford steers (230.4 ± 4.8 kg body weight [BW]) were sorted into 20 pens (2 steers/pen) following weaning (day 0). Treatments were randomly assigned to pens: (1) control: access to water only and (2) electrolyte: access to water and electrolyte solution supplementation (10% of total daily water intake) from days 1 to 14. Calf BW and blood samples were collected on days 0, 1, 3, 7, 14, 21, and 44 of the study. Blood samples were analyzed for sodium, potassium, albumin, haptoglobin, ceruloplasmin, and cortisol. All variables were analyzed using the MIXED procedure of SAS. Electrolyte solution consumption was estimated at 0.70 kg/calf daily (SEM ± 0.21). Calves assigned to the Electrolyte treatment had greater water and total liquid intake than control (*P* < 0.05). No effects of treatment were observed on ADG or BW (*P* > 0.05). Effects of day (*P* < 0.004), but not treatment or treatment × day were observed for sodium, potassium, albumin, cortisol, ceruloplasmin, and haptoglobin. Electrolyte solution supplementation during the preconditioning period did not improve performance nor influenced stress-related markers, however improved liquid intake.

## Teaser

Strategies to reduce stress and improve hydration after weaning and transportation are warranted. Electrolyte solution supplementation for beef calves during preconditioning increased water intake but did not lead to enhanced performance or attenuation of stress-related blood parameters.

## Introduction

Weaning and transportation are known as the major stressors in the production of beef cattle (Lynch et al., 2019). These challenging periods cause a disruption of homeostasis and induce adrenocortical and acute-phase responses, which are physiological mechanisms triggered to restore homeostasis (Chovatiya and Medzhitov, 2014). Although stress and inflammatory responses are protective reactions that act to preserve homeostasis, they can have adverse effects on the performance, immunity, and welfare of cattle (Carroll and Forsberg, 2007; Lynch et al., 2019). Additionally, behavioral responses such as reduced water and dry matter intake (DMI) are often observed in calves at weaning and shortly thereafter as a result of the insult (Weary et al., 2008; Overvest et al., 2018).

Changes in behavior and physiology can partially explain fluid loss in response to stress, particularly at weaning. Newly weaned calves are often challenged with exposure and adaptation to a new environment, which can make it difficult for them to achieve and maintain adequate water intake as they adjust to new surroundings (Enríquez et al., 2011). Moreover, physiologically, high cortisol levels can interfere with a principal mechanism of resistance to dehydration in cattle by increasing urination and reducing water intake (Parker et al., 2003). Although mechanisms are not entirely elucidated, this adrenocortical response contributes to aggravating dehydration and delays the return to homeostasis.

It has been shown that dehydration can cause significant changes in electrolyte balance (Schaefer et al., 1997), worsen existing diseases (Taylor et al., 2010), and increase disease susceptibility, morbidity, and mortality (Marcato et al., 2018; Renaud et al., 2018) in cattle. Therefore, finding strategies to alleviate stress, and the resulting dehydration observed during transitional and challenging periods is warranted to promote the welfare of cattle and to improve the efficiency of production.

Electrolyte solution supplied through drinkers (Knowles et al., 1997; Schaefer et al., 1997) or orally (Michell et al., 1992; Constable et al., 2001) can successfully replenish water and electrolytes, thereby improving health and performance of cattle. Calves treated with electrolytes have optimal recovery from diarrhea, dehydration, and moderate to severe acidosis (Smith, 2009). Schaefer et al. (1997) showed that cattle supplemented prior to slaughter with an electrolyte solution had greater hot carcass yield and less live weight loss when compared to cattle receiving just water. Additionally, fewer animals were classified as darker cutters when supplemented with an electrolyte solution. Similarly, Gortel et al. (1992) reported greater hot carcass yield and less live weight loss for cattle supplemented with an electrolyte solution prior to transport to a slaughter facility.

Electrolyte supplementation in beef cattle has been focused on feedlot animals, with supplementation primarily offered before transport and slaughter (Gortel et al., 1992; Schaefer et al., 1997, 2001). There is a paucity of published literature that has evaluated the use of electrolyte supplementation with different growth periods and during other challenging and stressful practices. Thus, the effects of using such technology with newly weaned calves in a preconditioning program are unknown.

We hypothesized that electrolyte solution supplementation of newly weaned beef calves during a preconditioning program would improve hydration status, attenuate stress-related physiological responses, and, consequently, enhance calf performance. Therefore, the objective of this study was to evaluate the effects of electrolyte solution supplementation on growth parameters and physiological markers associated with stress and acute-phase response of beef calves.

## Materials and Methods

All animal care and handling procedures were approved by the Institutional Animal Care and Use Committee of Oregon State University (IACUC-2022-0256). The study was conducted at the Eastern Oregon Agricultural Research Center—Oregon State University (EOARC, Burns, OR; 43°51ʹ86″N to 119°02ʹ15″W) from summer to fall (August to October) of 2022.

### Experimental Design and Calf Management

Forty Angus × Hereford steers (230.4 ± 4.8 kg body weight [BW], 195 ± 30 d of age) were enrolled in the study at weaning (day 0). Prior to study initiation, calves were maintained on a 6,500-ha semi-arid rangeland pastures at the Northern Great Basin Experimental Range (NGBER, Riley, OR; 43°29ʹ37″N to 119°42ʹ30″W) with their respective dams from turnout (summer grazing), when calves were ~30 ± 6 d of age, until weaning. Then, calves were weaned and transported ~76 km on a livestock trailer to the EOARC.

Upon arrival at EOARC, on day 0, calves were maintained as a single group and had free access to water and chopped (approximately 10 cm) alfalfa-grass hay. On day 1, calves were brought to the working facility, weighed, stratified by BW, and sorted into 20 pens (≥7 × 20 m; 2 steers/pen) for a 45-d preconditioning period. For the preconditioning period, treatments were randomly assigned to pens: (1) control: access to water only and (2) electrolyte: access to water and electrolyte solution.

Daily water intake (DWI) was estimated for all calves according to NASEM, (2016) and was offered to all calves daily in a galvanized steel tank (Behlen Country, 122 × 61 × 30.5 cm; 185 L max volume) placed within each pen. The DWI was estimated at ~35 L per calf based on the equation (Arias and Mader, 2011):


$$\begin{array}{l} DWI=-7.31+1.0\;\times DMI+0.04\;\times SR+0.3\;\times THI \end{array}$$

where DWI is the daily water intake (L/d), DMI is the dry matter intake (kg/d); SR is the solar radiation (W/m²), and THI is the temperature-humidity index.

Of the total expected DWI, 10% of water was fortified with the electrolyte supplement at a rate of 31.6 g/L (as per manufacture; TechMix; Stewart, MN). Calves assigned to electrolyte treatment had free access to the electrolyte solution from days 1 to 14 in an additional rubber tank (Rubbermaid, 66 × 66 × 25 cm, 19 L max volume) placed adjacent to the water tank in each pen. The electrolyte solution offered in each pen was individually weighed and mixed fresh daily before feeding (07:00 a.m.). The electrolyte supplement contained sodium chloride: 5.6% to 6.7% (minimum and maximum, respectively), sodium: 2.1% to 2.6% (minimum and maximum, respectively), potassium: 8.90% (minimum), zinc: 85 ppm (minimum) and naturally occurring microorganisms: Bacillus licheniformis and Bacillus subtilis: 89 million CFU/g (BlueLite 2Bw, TechMix; Stewart, MN). Overall, the same amount of liquid, was offered to both treatments without intake restriction.

All pens had concrete feed bunks (7 m) and automatic water tanks (Brower, 71 × 35.5 × 66 cm). During the supplementation period, automatic water tanks were turned off in order to properly evaluate water and electrolyte solution consumption. Electrolyte solution supplementation was provided from days 1 to 14, and thereafter electrolyte supplemental tanks and water tanks were removed. Thus, from days 15 to 45 calves had access to automated water tanks.

During preconditioning, calves received whole corn (0.9 kg/calf) and had ad libitum access to the chopped alfalfa-grass hay(14% and 59% crude protein [CP] and total digestible nutrients [TDN], respectively; 1.08%, 0.16%, 0.24%, 1.16%, 0.30% Ca, P, Mg, K, and Na, respectively; 705, 23, 7, 83, 0.80 ppm Fe, Zn, Cu, Mn, and Mo, respectively). Calves were fed in the morning, and feed intake was adjusted daily to allow minimal orts (target 100 g refusal/kg fed) without DMI restriction.

### Data and sample collections

Calf BW was collected at weaning (day 0) and on days 1, 14, 44, and 45. Calf BW on days 0 and 1 were averaged and considered calf weaning weight, whereas the final BW was obtained by the average of BW collected on days 44 and 45. Full BW was accessed instead of shrunk BW to minimize weight loss and avoid shrink-induced stress effects on blood parameters evaluated (Marques et al., 2012). Preconditioning average daily gain (ADG) was calculated by using weaning and final BW.

Blood samples were collected at weaning and on days 1, 3, 7, 14, 21, and 44 via jugular vein using commercial heparinized vacuum tubes for plasma harvest and tubes containing no additive for serum harvest (BD Vacutainer, 10 mL; Becton, Dickinson and Company, Franklin Lakes, NJ, USA). Blood samples were placed on ice immediately following collection and centrifuged at 2,500 × *g* for 30 min at 4 °C for serum and plasma harvest. Serum and plasma samples were frozen at −20 °C and stored at −80 °C until further analysis.

Consumption of water and electrolyte solution was monitored daily (days 1 to 14) by measuring the disappearance of water and electrolyte solution over a 24-h period. Intake was estimated as the amount of water or electrolyte solution offered minus refusals. After weighing refusals, clean water, and a newly mixed electrolyte solution were provided to each pen. Two identical water tanks to those used in the feedlot pens (one of each kind, steel, and rubber) were placed near the feedlot pens and filled with water and electrolyte solution to estimate evaporation losses, which were used to adjust water and electrolyte solution intake calculations.

Hay and corn samples were collected weekly, and composite samples were made for the entire preconditioning period. Samples were dried in a forced-air oven (55 °C) for 72 h, ground at 1- and 2-mm in a Wiley mill (model 3, Arthur H. Thomas, Philadelphia, PA, USA), and sent to a commercial laboratory (Dairy One Forage Laboratory, Ithaca, NY, USA) for nutrient analyses. Samples were analyzed by wet chemistry for CP (method 984.13; AOAC, 2006), neutral digestible fiber (Van Soest et al., 1991; modified for use in an Ankom 200 fiber analyzer, Ankom Technology Corp.) and TDN (Weiss et al., 1992). Results were 14% and 9% for CP, 53.3% and 10% for NDF, and 59% and 88% for TDN for the alfalfa-grass hay and corn samples, respectively. Feed refusals were removed and weighed daily, followed by weighing and offering the daily feed allocation. Feed intake was calculated on a DM basis by the difference in feed offered minus feed refusal.

### Environmental Data

Daily maximum, minimum temperatures, and relative humidity were collected from the National Weather Service, Boise, ID, Weather Forecast Office considering the nearest weather station available (Burns Municipal Airport Station, ~9 km from study location) from August to October of 2022.

Temperature and humidity parameters were used to calculate the THI and estimate potential heat stress (Dikmen and Hansen, 2009). The THI was calculated daily according to Mader et al. (2006):


$$\begin{array}{l} THI=(0.8\;\times T)+(\%RH\;÷100)\times (T-14.4)+46.4\; \end{array}$$

where T is the ambient temperature (°C) and RH is the relative humidity.

The THI values were interpreted according to the livestock weather safety index as follows: normal, ≤ 74; alert, 74 < THI < 79; danger, 79 ≤ THI < 84; and emergency, THI ≥ 84, and as reported by Mader et al. (2006) and Ranches et al. (2021a).

### Laboratory Analyses

Plasma samples were analyzed for cortisol, haptoglobin, and ceruloplasmin concentrations on days 0, 1, 3, 7, 14, 21, and 44; whereas serum albumin, Na, and K were analyzed on days 0, 1, 7, and 14. Cortisol, haptoglobin, and ceruloplasmin were evaluated as stress-related markers (Gomez-Laguna et al., 2011); whereas Na, K, and albumin concentrations were measured as an indicator of hydration status (Parker et al., 2003; Averós et al., 2008).

Plasma haptoglobin concentrations were determined in duplicate samples by a biochemical assay measuring the haptoglobin–hemoglobin complex by estimating differences in peroxidase activity (Makimura and Suzuki, 1982). Results were obtained as arbitrary units resulting from reading plates at 450 nm (VersaMax Tunable EXT). The same quality control standards used in the biochemical assay were analyzed by quantitative determination of bovine haptoglobin in plasma (bovine haptoglobin ELISA test kit; Life Diagnostics, Inc., West Chester, PA, USA). The concentration of haptoglobin, based on the ELISA assay, ranged from 0.03 (low control) to 0.95 mg/mL (high control) with an intra-assay coefficient of variability (CV) of 1.26%. The ELISA standard curve was used to convert the arbitrary units obtained from the biochemical procedures into mg/mL (Cooke and Arthington, 2013) with the least detectable value of 0.03 mg/mL. The intra- and inter-assay CVs were 3.1% and 9.3%, respectively.

Plasma ceruloplasmin oxidase activity was measured in duplicate samples using colorimetric procedures described by Demetriou et al. (1974). Ceruloplasmin concentrations are expressed as milligrams per deciliter as described by King (1965). The intra- and inter-assay CVs were 3.7% and 11.1%, respectively.

Plasma cortisol concentrations were measured using chemiluminescent enzyme immuno-assays (Immulite 1000; Siemens Medical Solutions Diagnostics, Los Angeles, CA) with an intra-assay CV of 4.6%.

Serum albumin concentrations were determined with a commercial Enzyme-linked immunosorbent assay kit (Mybiosource Inc., San Diego, CA) according to the manufacturer’s protocol. The intra- and inter-assay CVs were 4.2% and 4.0%, respectively.

Serum Na concentrations were measured with commercial kits (Crystal Chem Inc., Elk Grove Village, IL) based on an enzymatic method using Na-dependent β-galactosidase activity with *ortho*-nitrophenyl-β-galactosidase as substrate. The intra- and inter-assay CVs were 6.5% and 10.8%, respectively. Serum K concentrations were measured based on a kinetic coupling assay system using K-dependent pyruvate kinase with commercial kits (Crystal Chem Inc., Elk Grove Village, IL). The intra- and inter-assay CVs were 6.1% and 7.3%, respectively.

### Statistical Analyses

All data were analyzed using the MIXED procedure of SAS (SAS Inst. Inc., Cary, NC; Version 9.4) in a complete randomized design considering pen as the experimental. Water intake, total liquid intake, and DMI variables were analyzed with pen(treatment) as a random effect, whereas blood parameters, BW, and ADG variables were analyzed considering pen(treatment) and calf(pen) as a random effect. Blood parameters and water intake were evaluated as repeated measurements with calf(pen) and pen(treatment) as subjects, respectively, and the best structure of covariance matrix was chosen based on the smallest Akaike’s information criterion with correction. Electrolyte solution intake was evaluated within the Electrolyte treatment as repeated measures over time. Compound symmetry was used as the covariance structure for all repeated measures as it produced the lowest Akaike’s information criterion. Variables were tested for the fixed effects of treatment, day, and treatment and day interaction. Least square means for DMI, DWI, and electrolyte solution intake were reported as average per pen. Significance was set at *P* ≤ 0.05, and tendencies if *P* > 0.05 and ≤ 0.10.

## Results and Discussion

**Figure 1.**
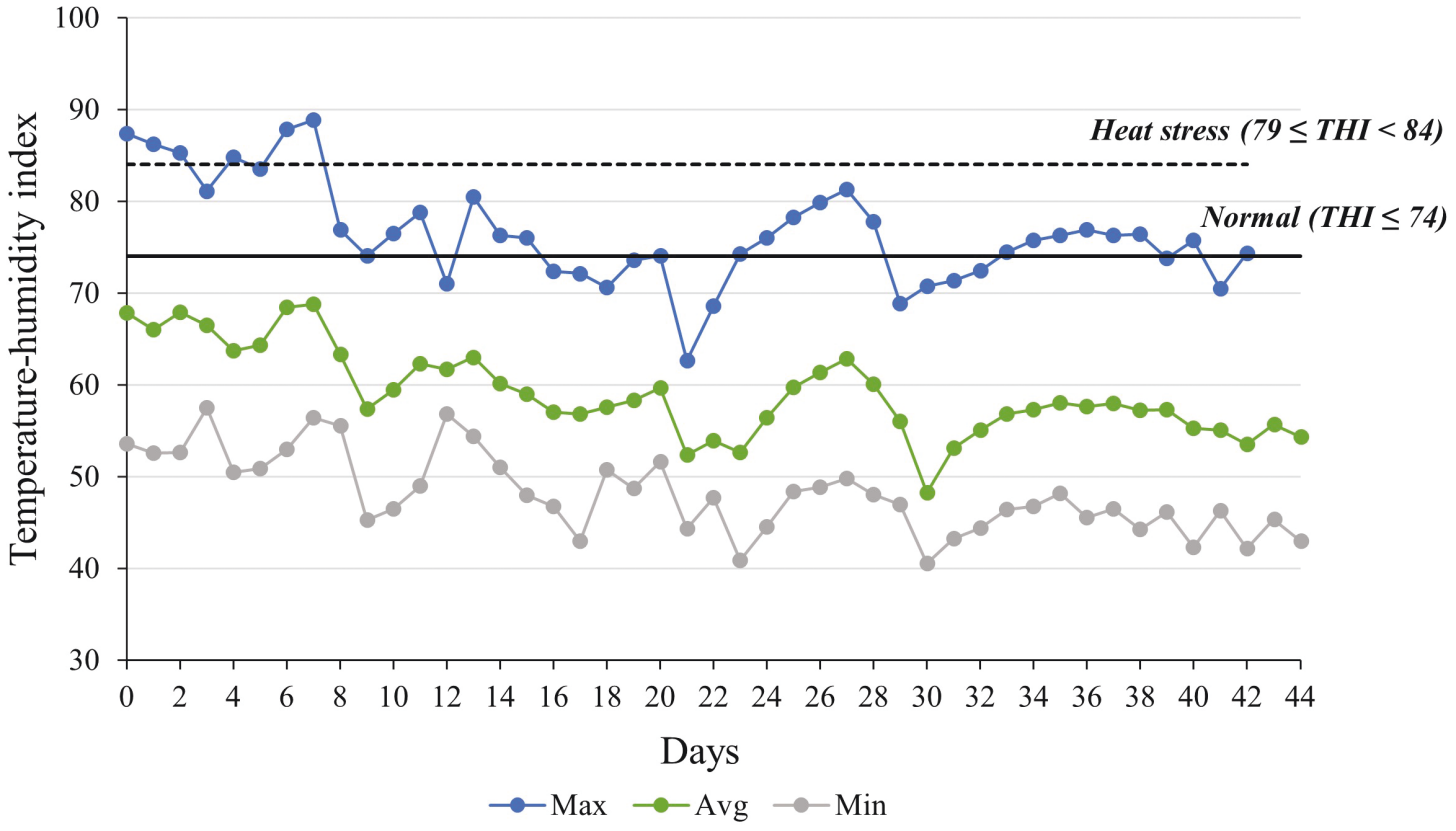
Temperature-humidity index (THI) was calculated throughout the study based on temperature and relative humidity. Max, maximum; Avg, average; Min, minimum.

Average, maximum, and minimum THI throughout the study were 59.2, 76.5, and 48.3, respectively (Figure 1). Calculating THI provides valuable and additional information on variables that could affect hydration and, consequently, aggravate stress-related responses (Mader et al., 2006). The average THI values observed throughout the study were below the threshold set as normal for heat stress (<74 THI), which means that, although the study was conducted during a time that typically coincides with elevated environmental temperatures, the calves in our study were in a thermoneutrality state. This is likely explained by the large thermic amplitude observed in the Great Basin area (National Weather Service, 1995).

**Figure 2.**
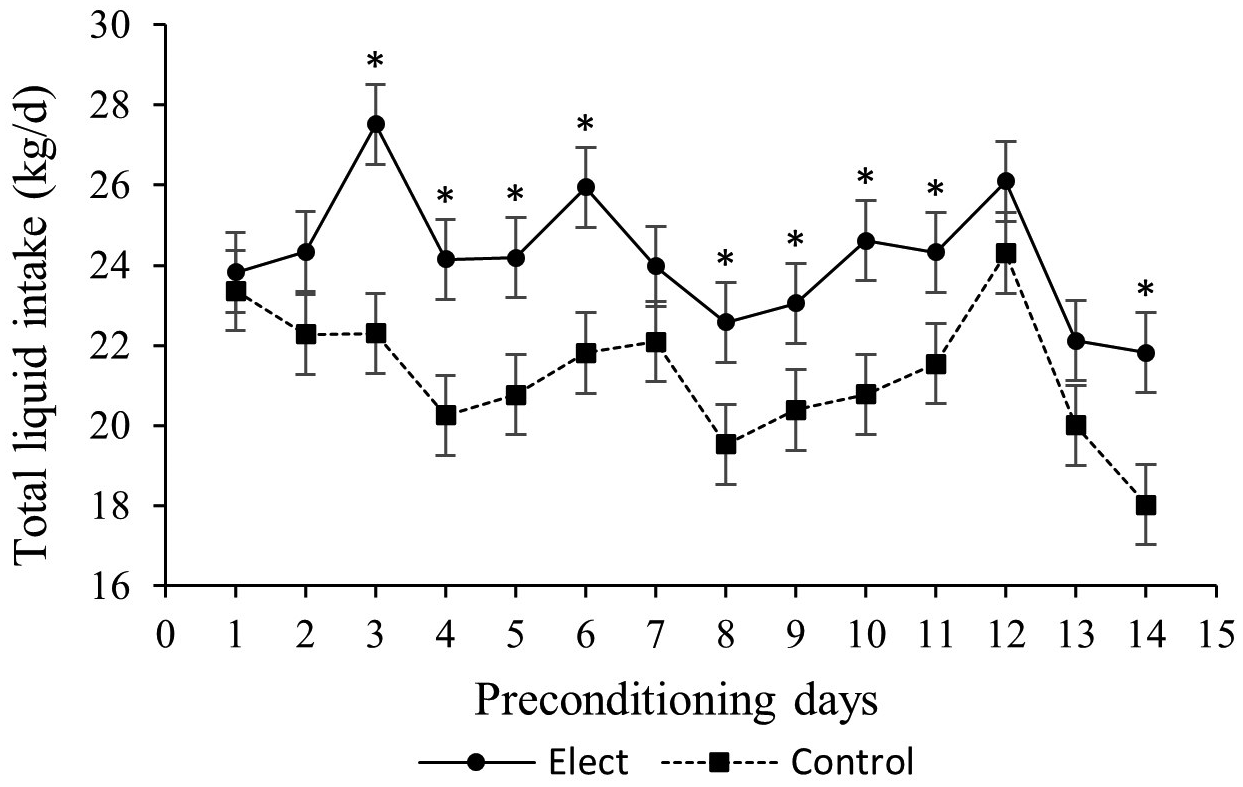
Total liquid intake of calves receiving water or electrolyte solution supplementation (water plus electrolyte solution) for 14 d during preconditioning. ^*^Means differ between treatments (*P <* 0.05).

Effect of treatment (*P* = 0.01) and day (*P* < 0.001) was found for DWI and total liquid intake (DWI plus electrolyte solution; Table 1). Calves assigned to electrolyte treatment had on average, 10% greater DWI compared to calves assigned to the control treatment (Table 1). A tendency for a treatment × day interaction (*P* = 0.08) was found for total liquid intake (Table 1), where calves assigned to electrolyte treatment had greater total liquid intake from days 3 to 11 when compared to calves assigned to the control treatment (*P* < 0.05; Figure 2).

**Table 1.** Effect of an electrolyte solution on liquid intake of calves during preconditioning

	Treatments^1^			*P*-value		
Item	Control	Electrolyte	SEM	Treatment	Day	Trt × Day
Water intake, kg/calf/d	21.3	23.5	0.541	0.01	< 0.0001	0.20
Total liquid intake, kg/calf/d^2^	21.3	24.2	0.549	0.001	< 0.0001	0.08
Electrolyte solution intake, kg/calf/d	—	0.70	0.202	—	0.31	—

^1^Control: water; electrolyte: water and an electrolyte solution (BlueLite 2Bw, TechMix; Stewwart, MN, USA) provided daily on one additional tank per pen. Approximately 10% of the expected daily water intake was fortified with the electrolyte solution at a rate of 31.6 g/L. Assigning weaning as day 0, electrolyte solution supplementation was provided from days 1 to 14 during preconditioning.

^2^Total liquid intake—water plus electrolyte supplement intake.

The NASEM (2016) presents an estimation for beef cattle water intake based on the study by Winchester and Morris (1956), using BW and an effective temperature index. In this study, calves assigned to control and electrolyte treatments consumed an average of 21.1 and 24.2 L of total liquid intake, respectively, which was within the range of 20 to 48 L/calf/d suggested by NASEM (2016) according to changes in temperature (4.4 to 32.2 °C for a 273 kg growing calf). The effect of day on water consumption is likely related to natural fluctuations of intake and temperature throughout the study, other than the effect of weaning and transportation. According to Ahlberg et al. (2018), temperature is responsible for 19.4% of water intake variation for cattle, while DMI seems to have less impact on water intake compared to ambient temperature (NASEM, 2016).

Calves assigned to electrolyte treatment consumed 0.7 kg (SEM ± 0.21) of electrolyte solution daily, which consisted of 20% of the offered amount (Table 1). The reasons for calves not consuming as much of the electrolyte solution as expected are currently unknown. It is possible that the opportunity to choose between water and electrolyte solution had an impact on the electrolyte solution consumption as calves were not familiar with the electrolyte solution and could, therefore, favor water consumption. Additionally, optimal supplementation rates with electrolyte solutions are not fully understood, and further investigation evaluating voluntary intake is necessary to determine the optimal supplementation rate for calves at this developmental stage and in this scenario. Further, calves were transported for a relatively short distance and processed in facilities previously known to them under minimal stress. Thus, water as the main source of hydration was likely enough to sustain physiological requirements and homeostasis. Moreover, THI levels throughout the study (Figure 1) show that calves were in a comfort zone (Mader et al., 2006) and that temperatures were mild and favorable for rapid recovery from weaning and transportation.

Although calves assigned to electrolyte treatment did not consume the expected amount of electrolyte solution (which was 10% of the expected total water intake), it is possible that providing the electrolyte solution may have attracted the calves more often to water tanks, as tanks were in close proximity, resulting in greater water consumption to calves assigned to electrolyte treatment when compared to calves assigned to control treatment. Similarly, Smithyman et al. (2022) reported that newly received heifers that were offered an electrolyte solution for 3 d after arrival at the feedlot showed greater water intake compared to those offered only water. Nonetheless, consistent with the findings of this study, the increase in water intake did not translate into any improvement in the performance or blood parameters of the calves.

No effects of treatment (*P* > 0.05) were observed on BW, ADG, and DMI for any period (Table 2). The lack of differences in performance may be attributed to the low consumption of electrolyte solution by the calves. Previous studies supplementing electrolyte solution before slaughter have identified differences in the BW of bulls with a consumption of 2.4% to 3.0% of BW of electrolyte solution (Gortel et al., 1992; Schaefer et al., 1997). Authors suggest that these differences are not solely related to gut fill or hydration but rather suggest retention of electrolytes in the muscle tissue. In the current study, calves assigned to electrolyte treatment had access to both water and electrolyte solution and consumed approximately 0.3% of BW of electrolyte solution. This indicates that the calves may not have consumed enough of the electrolyte solution to have an optimal effect on performance and physiology.

**Table 2.** Effect of an electrolyte solution on body weight, average daily gain, and dry matter intake of calves during preconditioning

	Treatments^1^		SEM	*P*-value
Item^2^	Control	Electrolyte		
Initial BW, kg	230.5	230.3	4.79	0.97
Period: days 1 to 14				
Day 14 BW, kg	251.8	251.5	5.454	0.96
ADG, kg/d	1.61	1.65	0.108	0.78
DMI, kg/calf/d	4.65	4.66	0.091	0.92
Period: days 14 to 44				
Day 44 BW, kg	267	265	5.363	0.81
ADG, kg	0.45	0.52	0.051	0.33
DMI, kg/calf/d	5.75	5.91	0.275	0.42
Overall				
ADG, kg	0.83	0.80	0.050	0.61
DMI, kg/calf/d	5.38	5.50	0.216	0.48

^1^Control: water; electrolyte: water and an electrolyte solution (BlueLite 2Bw, TechMix; Stewwart, MN, USA) provided daily on one additional tank per pen. Approximately 10% of the expected daily water intake was fortified with the electrolyte solution at a rate of 31.6 g/L. Assigning weaning as day 0, electrolyte solution supplementation was provided from days 1 to 14 during preconditioning.

^2^BW, body weight; ADG, average daily gain; DMI, dry matter intake.

No effects of treatment or treatment × day interaction were observed for cortisol (*P* > 0.05), ceruloplasmin (*P* > 0.05), and haptoglobin (*P* > 0.05; Table 3); however, an effect of day was found for these variables (*P* < 0.001; Table 4).

**Table 3.** Effect of an electrolyte solution on blood metabolites and acute-phase response markers of calves during preconditioning

	Treatments^1^			*P*-value		
Item	Control	Electrolyte	SEM	Treatment	Day	Trt × Day
Sodium, mmol/L	123	121	2.573	0.64	< 0.0001	0.62
Potassium, mmol/L	5.52	5.36	0.163	0.52	< 0.0001	0.31
Albumin, g/dL	5.31	5.30	0.171	0.95	0.004	0.87
Cortisol, ng/mL	2.25	2.35	0.147	0.64	< 0.0001	0.86
Haptoglobin, µg/mL	0.63	0.66	0.029	0.57	< 0.0001	0.99
Ceruloplasmin, mg/mL	27.5	26.4	0.659	0.29	< 0.0001	0.14

^1^Control: water; electrolyte: water and an electrolyte solution (BlueLite 2Bw, TechMix; Stewwart, MN) provided daily on one additional tank per pen. Approximately 10% of the expected daily water intake was fortified with the electrolyte solution at a rate of 31.6 g/L. Assigning weaning as day 0, electrolyte solution supplementation was provided from days 1 to 14 during preconditioning.

**Table 4.** Blood metabolites and acute-phase response markers of calves during preconditioning

	Preconditioning days^1^								*P*-value
Item	Day 0	Day 1	Day 3	Day 7	Day 14	Day 21	Day 44	SEM	Day
Sodium, mmol/L	122.1^a^	110.5^b^	—	124.1^a^	130.9^a^	—	—	0.243	< 0.0001
Potassium, mmol/L	5.84^a^	4.62^c^	—	6.15^a^	5.15^b^	—	—	3.422	< 0.0001
Albumin, g/dL	5.12^b^	5.91^a^	—	4.90^a^	5.28^a^	—	—	0.210	0.004
Cortisol, ng/mL	2.20^b^	2.61^a^	2.08^b^	1.96^b^	2.05^b^	2.20^b^	2.18^b^	0.183	< 0.0001
Haptoglobin, µg/mL	0.43^c^	1.14^b^	1.55^a^	0.34^c^	0.33^c^	0.38^c^	0.37^c^	0.075	< 0.0001
Ceruloplasmin, mg/mL	27.5^b^	26.5^b^	31.0^a^	30.9^a^	30.8^a^	19.7^d^	22.1^c^	0.801	< 0.0001

^a-d^Within a row, different superscripts show differences (*P* ≤ 0.05) between days.

^1^Assigning weaning as day 0, treatments were provided from days 1 to 14 during preconditioning.

Plasma cortisol concentration peaked on day 1 (*P* < 0.001) and then decreased and remained stable throughout the study. Similarly, plasma haptoglobin concentration was greater on day 3 followed by day 1 (*P* < 0.001; Table 4), and thereafter concentration was maintained for the remainder of the study. Plasma ceruloplasmin concentrations were greatest on days 3, 7, and 14 (*P* < 0.001), and decreased on days 21 and 44 (*P* < 0.001; Table 4).

Cortisol and the acute-phase proteins (haptoglobin and ceruloplasmin) were evaluated as weaning stress markers, and the results observed were consistent with other work evaluating stress postweaning (Arthington et al., 2005, 2008; Ranches et al., 2021b). Cortisol plays a role in regulating the process of stress adaptation by stimulating nutrient mobilization from the liver, fat, and muscle (Ralph and Tilbrook, 2016). This catabolic effect contributes to increasing the availability of circulating nutrients, enabling the animal to effectively restore homeostasis. However, while cortisol-induced nutrient mobilization is a crucial aspect of stress adaptation, it also triggers a complex cascade of events within the immune system. Immune cells perceive tissue mobilization as a deviation from homeostasis and initiate the production of cytokines, which are the main stimulators of acute-phase protein production in the liver (Carroll and Forsberg, 2007).

Stress can also induce dehydration and electrolyte imbalance, thus impacting both animals’ health and welfare (Schaefer et al., 1997). Although mechanisms are not fully understood, cortisol seems to interfere with a principal mechanism of resistance to dehydration in cattle (Parker et al., 2003, 2004) via inhibition in the action of the antidiuretic hormone, arginine vasopressin. Studies have shown that high cortisol levels can increase urination and reduce water intake in cattle and sheep (Parrott et al., 1987; Parker et al., 2003). Therefore, in addition to the typical decrease in DMI and water consumption following weaning and transportation, the adrenocortical response can aggravate dehydration. The initial assumption of this study was that electrolyte solution supplementation would promote a quicker return to homeostasis and improve hydration compared with non-supplemented calves, leading to a better response to weaning stress. According to Schaefer et al. (2001), electrolyte supplementation was an efficacious way to mitigate stress prior to slaughter in market cattle, but no physiological markers were evaluated in that study. Previous work has shown that few differences were observed in blood parameters between cattle given or not given electrolyte solution (Schaefer et al., 1992; Smithyman et al., 2022). Studies have reported no differences in glucose, beta-hydroxide butyrate, hematocrit, or plasma electrolytes of bulls receiving water or electrolytes prior to slaughter (Gortel et al., 1992; Schaefer et al., 1992). To the best of our knowledge, this is the first study that has evaluated the effect of an electrolyte solution on stress markers and acute-phase proteins during preconditioning.

An effect of the day (*P* < 0.004; Table 3), but not treatment or treatment × day, was observed for albumin, Na, and K. On day 1, there was an increase in serum albumin concentration (*P* < 0.001), which then returned to the same concentration as observed on days 0, 7, and 14 (Table 4). Conversely, Na serum concentration was reduced on day 1, and K concentration was reduced on day 1 followed by day 14 (*P* < 0.001; Table 4). Similarly, to this study, others have also found no changes in Na and K serum concentrations in cattle offered electrolyte supplementation (Schaefer et al., 1997).

The reduced serum K concentration, and increase in albumin on day 1 was likely a consequence of dehydration after weaning and transportation. On the other hand, reduced concentration of sodium on day 1 is discordant since it has been shown that water deprivation causes an increase in sodium serum concentrations (Parker et al., 2003).

Albumin is a protein primarily produced in the liver that binds metabolites and regulates plasma osmolality (Quinlan et al., 2005). Dehydration leads to a decrease in total body water, resulting in a higher concentration of solutes in the bloodstream, including albumin. This relative increase occurs as the volume of plasma decreases due to the loss of water. The observed increase in albumin on day 1 in this study is consistent with previous findings that showed increased serum albumin concentration in cattle after weaning and transport, suggesting dehydration may have occurred (Kent and Ewbank, 1986; Averós et al., 2008). However, the slightly greater water consumption observed for calves assigned to electrolyte did not contribute to changes in serum albumin concentration and therefore albumin concentration returned to nadir. It is important to note that albumin is just one marker of complex physiological indicators of dehydration, and other parameters, such as electrolyte concentration and hematocrit, should be considered to obtain an accurate assessment of hydration status in cattle.

Discussion of literature that utilized animals in different conditions or stages of production was made due to the lack of prior research on calves after weaning. It must be considered that the effectiveness of providing electrolytes to cattle under water withdrawal associated with long-distance transportation would indeed be greater compared to recently weaned calves. In addition, the low consumption of the electrolyte solution by the calves may have been the primary factor contributing to the absence of differences in performance and physiological responses as initially anticipated.

Therefore, differences between this study and previous studies could be due to variations in the age and animal category, length of supplementation, the severity of the stress period, length of water deprivation, and dehydration status.

## Conclusion

Overall, providing an electrolyte solution to preconditioning calves increased water intake but did not lead to enhanced performance or attenuation of stress-related blood parameters. Further investigation is needed to determine the optimal consumption of electrolyte solution and timing of supplementation to improve calf performance and physiological responses to stress during the preconditioning phase.
